# 5,10-methylenetetrahydrofolate reductase C677T gene polymorphism as a risk factor for premature coronary artery disease in patients with type 2 diabetes mellitus

**DOI:** 10.3389/fendo.2024.1502497

**Published:** 2025-01-22

**Authors:** Nisreen O. Mohammed, Ibtisam A. Ali, Bahaelddin K. Elamin, Bakri Osman Saeed

**Affiliations:** ^1^ Ahfad Centre for Science and Technology, Ahfad University for Women, Khartoum, Sudan; ^2^ Faculty of Medicine, International University of Africa, Khartoum, Sudan; ^3^ Department of Medical Microbiology, Faculty of Medical Laboratory Sciences, University of Khartoum, Khartoum, Sudan; ^4^ Faculty of Medicine, Sudan International University, Khartoum, Sudan

**Keywords:** diabetes mellitus, hyperhomocysteinemia, MTHFR, CAD, polymorphism

## Abstract

**Background:**

Africa, like the rest of the world, is experiencing an increasing prevalence of diabetes mellitus. Diabetes increases the risk for coronary artery disease (CAD) by fourfold compared to people without diabetes. C677T polymorphism in methylenetetrahydrofolate reductase (MTHFR) and hyperhomocysteinemia were reported by many studies as risk factors for CAD among patients with type 2 diabetes mellitus (T2DM). Early detection of modifiable risk factors for CAD is an important aspect of management of diabetes. This is the only study in Sudan which investigates the association between MTHFR genotypes and plasma homocysteine levels, and their role in premature CAD (PCAD) among patients with T2DM.

**Methods:**

This study is a comparative study. We enrolled 226 Sudanese patients with T2DM, age range 25-60 years, recruited from Alshaab and Omdurman teaching hospitals in Khartoum State. 113 patients had CAD confirmed by angiography and electrocardiography (ECG) and 113 had no evidence of CAD. Polymerase chain reaction (PCR) and restriction fragment length polymorphism (RFLP), using Hinf1 restriction enzyme, were used to determine MTHFR genotypes. Plasma homocysteine levels were determined by enzymatic assay on the Hitachi Cobas Integra^®^ 400 plus. Data was analyzed using statistical package for Social Sciences (SPSS) 23, using Mann-Whtney U test, general linear model, Chi-square test and logistic regression analysis.

**Results:**

The frequencies of TT, CT, and CC genotypes were 16,40 and 44% among T2DM patients with PCAD. In T2DM patients without PCAD, the frequencies of TT, CT, and CC genotypes were 00,19 and 83%. The T allele showed strong association with PCAD among T2DM patients, *p <*0.001, odds ratio (OR) 6.2, 95% CI (3.4-11.6). Patients with PCAD showed higher plasma homocysteine levels than patients without PCAD (13.5 µmol/L versus 10 µmol/L, *p* < 0.001). The T allele had significant effect on homocysteine level, (*p <*0.001). Plasma homocysteine levels were higher in individuals with TT genotype than those with CT or CC genotypes in patients with PCAD (16.2 + 5.3, 14.3 + 5.7 and 12.9 + 5.02 µmol/L, *p*=0.017). Homocysteine levels showed a significant association with CAD, *p*<0.001, OR 3.2, 95% CI (1.9—5.5).

**Conclusions:**

Our study suggests that C677T polymorphism of MTHFR gene and hyperhomocysteinemia are risk factors for PCAD in Sudanese population with T2DM.

## Introduction

Globally, the burden of diabetes mellitus (DM) continues to increase. The International Diabetes Federation (IDF) estimates that in 2022, 537 million adults live with diabetes worldwide (1).

Africa, like the rest of the world, is experiencing an increasing prevalence of DM, 1 in 22 adults live with DM in Africa (1, 2). In Sudan, previous studies have shown that there is high prevalence of diabetes in the adult populations (1, 3). The IDF reported, in 2021, that Sudan is one of the top five countries in the prevalence of diabetes in the Middle East and North Africa (4).

Diabetes increases the risk for coronary artery disease (CAD) by fourfold compared to people without diabetes (5, 6). CAD leads to significant morbidity and mortality. Identification and management of risk factors for CAD is an important aspect of management of DM.

Hyperhomocysteinemia has been identified as an independent risk factor for CAD (7, 8). Hyperhomocysteinemia has been associated with genetic defects in enzymes involved in its metabolism and/or with nutritional deficiencies of vitamin B_6_, B_12_ and folic acid (9, 10). Numerous studies have documented the influences of a common polymorphism (C677T) of methylenetetrahydrofolate reductase (MTHFR) on homocysteine levels. However, the relationship between this mutation and cardiovascular diseases (CVD) has remained a controversial issue (11–13).

Homocysteine and diabetes appear to have a negative synergistic effect on the cardiovascular system, and it is therefore important to investigate this effect in patients with diabetes who have higher risk for CAD than the normal population (14).

Homocysteine (Hcy) is metabolized by either remethylation to methionine or transsulphuration to cysteine. The former reaction is catalyzed by the vitamin B_12_-dependent methionine synthase. The transsulfuration pathway is catalyzed by cystathionine-β-synthase (CβS) which is a vitamin B6 dependent process (13, 15).

MTHFR catalyzes the reduction of 5,10-methylene tetrahydrofolate to 5 methyltetrahydrofolate, which is the methyl donor in the remethylation of Hcy to methionine. The most common mutation in the MTHFR gene is the 677_→_T variant which has been shown to encode a thermolabile enzyme with reduced activity (16, 17).

Several studies investigated possible association between MTHFR genotypes and plasma homocysteine levels and the incidence of different MTHFR genotypes in CAD patients (18–22). The results of these studies have been controversial. Many studies failed to show association between MTHFR genotypes and plasma homocysteine levels and their role in CAD. The variation was thought to be caused by ethnic or geographical differences, at least in some of these studies (23–25).

There is paucity in the studies which included patients with diabetes, who are already at increased risk for CAD (2, 5).

In Sudan, there is a gap in our knowledge about the most important modifiable risk factors for PCAD; therefore, identifying these factors in our population can improve prevention of these serious diabetic complications.

In this study, we investigated the association between MTHFR genotypes and plasma homocysteine (Hcy) levels and their role in PCAD in Sudanese patients with type 2 diabetes mellitus.

## Materials, methods and study subjects

This is a comparative study, using a non-probability, selective sampling technique. 226 patients with diabetes were enrolled in this study according to statistical power formula.

113 patients had CAD and 113 had no evidence of CAD.

CAD was confirmed by angiography (≥ 50% stenosis of at least one of coronary arteries) and electrocardiography (ST segment depression or ST segment elevation) (26).

T2DM was diagnosed according to the WHO criteria (Glycated hemoglobin (HbA1c), ≥ 6.5%, 48 mmol/mol) or fasting plasma glucose ≥7.0 mmol/L (126 mg/dL) (27). Type1diabetus mellitus patients (T1DM) were excluded from the study. None of our patient was on insulin treatment. The age range of our study population was 25-60 years. The study was conducted in 2 hospitals (Alshaab and Oumdurman teaching hospitals) in Khartoum State, which treats Sudanese patients from different ethnic backgrounds.

Smoking was defined as the use of any tobacco at the time of conducting the study, while hypertension was defined with as systolic blood pressure >130 mmHg and diastolic blood pressure > 90 mmHg or using anti-hypertensive medication. The study protocol was approved by the Sudanese ministry of health ethics committee. Informed consent was obtained from all patients prior to data collection; investigation was conducted in accordance with the Declaration of Helsinki.

### Biochemical analysis

Venous blood was obtained in Ethylenediamine tetraaetate (EDTA) after an overnight fast. Plasma Hcy level was determined by enzymatic assay on Hitachi Cobas Integra^®^ 400 plus, the principle of the test is based on measuring the co-substrate conversion product, NAD spectrophotometrically at 340 nm. The lipid profile was estimated using Roche Cobas c311 based on enzymatic method. Glycated hemoglobin (HbA1c) was measured by a turbidimetric inhibition immunoassay (TINA) using Roche Cobas c311 analyzer. Authentic reagents were obtained from Roche diagnostics dealer in Sudan. The biochemical analysis was performed in East Model hospital in Khartoum.

### DNA preparation and genotyping

The PCR procedure was performed in the centre of Sudanese association for supporting patients with kidney disease.

Buffy coat was used for DNA extraction following the manufacturer’s guide of QIAquick Gel Extraction Kit (1000) From QIAGEN (Manchester, United Kingdom). The MTHFR C677T single-nucleotide polymorphism (SNP) was determined by polymerase chain reaction-restriction fragment length polymorphism (PCR-RFLP) method using the Hinf I restriction endonuclease enzyme (BioLabs, Frankfurt, Germany). The primers used were forward (5’- TGA AGG AGA AGG TGT CTG CGG GA -3’, and reverse, 5’- AGG ACG GTG CGG TGA GAG TG -3’). The PCR mixture used contained 18.5 µl 5xFIREPoL ^®^ Master Mix from SOLIS BIODYNE company, Tartu, Estonia, 5 µl of DNA template and 0.5µl of each primer were added.

PCR was performed under the following cycling conditions: 94°C for 3 min (initial denaturation), followed by 94°C for 1 min, 61°C for 1 min (annealing), 72°C for 2 min, and 72°C for 3 min (final extension), for a total of 40 cycles using MULTI GENE OPTI MAX from Cleaver Scientific Ltd (Rugby, United Kingdom).

PCR products were electrophoresed on agarose gel (2%, 0.5 mg/mL ethidium bromide) and visualized using Gel documentation system CSL-MICRODOC System from Cleaver Scientific (Rugby, United Kingdom).

PCR-RFLP was used to determine the MTHFR C677T genotypes. PCR products were subjected to overnight restriction digestion by HinfI (5Units) at 37°C. Restriction products were electrophoresed on agarose gel (2%, 0.5 mg/mL ethidium bromide) and visualized as was the PCR product.

**Figure 1:**
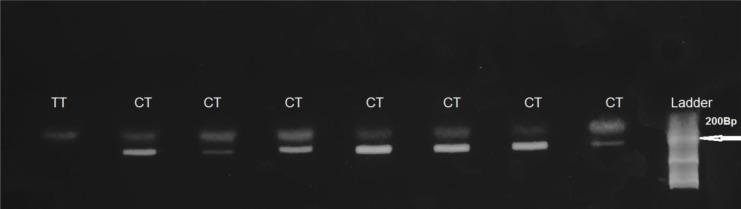
HinfI-treated MTHFR gene PCR fragments separated on 2% agarose gel. Presence of the C-allele resulted in no cleavage of the PCR product. Heterozygote C677T, (CT genotype) yielded two fragments of 198 bp and 175 bp. Homozygote C677T (TTgenotype) yielded two fragments of 175 bp.

Presence of the C-allele resulted in no cleavage of the PCR product. Heterozygote C677T yielded two fragments of 198 bp, 175 bp. Homozygote C677T yielded two fragments of 175 bp (**Figure 1**).

### Statistical analysis

The data collected was analyzed using Statistical Package for the Social Sciences (SPSS) 23. Categorical variables were expressed as numbers and percentages. Mann-Whitney-U test was used to compare differences between quantitative variables among the two groups and expressed as median and Interquartile Range (IQR), general linear model was used to determine the significant differences in homocysteine levels according to genotypes, chi square test was used to detect the association of MTHFR polymorphisms with CAD and logistic regression analyses was used to test the association between (selected significant independent variables) and CAD among T2DM patients, *p* < 0.05 was considered statistically significant.

## Results

The clinical and biochemical characteristics of patients were summarized in **Table 1**.

**Table 1:** Clinical and biological characteristics of diabetic patients with and without CAD.

Variables	T2DM with CAD (n=113)	T2DM without CAD (n=113)	*p* value
Sex (Male/female)	70/43	63/50	0.3
Smoking n (%)	37(33%)	14 (12%)	< 0.001
Age/year’s	50 (42—55)	51(48—55)	0.02
Duration of Diabetes/Years	7 (3.0—10)	6 (1.0—8.0)	0.02
Duration of Hypertension/Years	10 (6.0—12.0)	7 (5.0—10.0)	0.01
Cholesterol level	3.6 (2.9—4.9)	3.8 (3.3—4.3)	0.7
Triglyceride level	1.2 (1.01—1.5)	0.9 (0.8—1.7)	< 0.001
LDL Cholesterol	0.8 (0.7—1.1)	1.0 (1.23—1.1)	< 0.001
HDL cholesterol	0.9 (0.8—1.01)	1.1 (0.9—1.3)	< 0.001
Non-HDL cholesterol	2.5 (2.01—3.9)	2.5 (2.3—3.2)	0.5
HbA1c	75 (64—86)	75 (53—86)	0.4
Hcy level	13.5 (11.0—18.3)	10 (9.0—15)	< 0.001

All quantifiable parameters are reported as median and interquartile range (IQR). IQR is the distance between the 25^th^ (Q1) and 75^th^ (Q3) percentiles.

Lipid levels are in millimoles per liter (mmol/L). HbA1c levels are in millimoles per mole (mmol/mol). Homocysteine levels are in micromoles per liter (µmol/L).

Gender had no significant effect on CAD, *p* > 0.05(0.3). Age, smoking, duration of diabetes and hypertension were significantly different between the two groups, *p* < 0.02, < 0.001, 0.02 and 0.01 respectively, Diabetic patients with CAD have significantly higher levels of plasma triglycerides, LDL cholesterol and lower levels of HDL cholesterol, *p* < 0.001 but no difference noted in total cholesterol, and non-HDL cholesterol levels between the two groups, *p* > 0.05, 0.7 and 0.5 respectively. Plasma homocysteine levels were significantly higher in diabetic patients with P CAD, *p* < 0.001.

### Association of MTHFR gene polymorphisms with PCAD in patients with T2DM

The frequencies of TT, CT and CC genotypes among patients with T2DM and CAD were 16, 40 and 44%, and were 00, 19 and 83% in T2DM patients without CAD, (*p* < 0.001). The frequency of the T allele was higher in patients with PCAD than without CAD (0.36 versus 0.08%, *p* < 0.001). The odds ratio (OR) for CAD in T2DM patients who carry the T allele was 6.2, CI 95% (3.4-11.6) (**Table 2**).

**Table 2:** Distribution of MTHFR genotypes and alleles in type 2 diabetic patients with and without CAD.

	T2DM with CAD (n=113)	T2DM without CAD (n=113)	*p* value
Genotypes			
CC	50 (44%)	94 (83%)	< 0.001
CT	45 (40%)	19 (17%)	
TT	18 (16%)	00(0%)	
Allele			
C	0.64	0.92	< 0.001
T	0.36	0.08	

C is the wild-type allele and T is the mutant allele.

CC genotype is homozygous for the wild-type C677 allele, CT genotype is heterozygous for the mutant 667T allele and TT genotype is homozygous for the mutant 667T allele.

### Correlation between MTHFR genotypes and plasma homocysteine levels

Plasma homocysteine levels were significantly different between MTHFR genotypes:

16.2 ± 5.3, 14.3 ± 5.7 and 12.9 ± 5.02 µmol/L in TT, CT and CC genotypes respectively, *p* = 0.017. *Post hoc* analysis showed significantly higher levels of homocysteine in the TT genotype than CC genotype, *p* = 0.03. Homocysteine levels showed significant association with CAD, *p* < 0.001, OR 3.2, 95% CI (1.9-5.5), (**Table 3**).

**Table 3:** Effect of MTHFR genetic polymorphism on homocystiene levels.

Genotypes	Homocystiene Levels (µmol/L) ± SD	*p*. value
CC	12.9 ± 5.02	< 0.05(0.017) *
CT	14.3 ± 5.7	
TT	16.2 ± 5.3	

*General linear model showed a significant difference in homocysteine levels between the three genotypes, p = 0.017, and post Hoc analysis showed a significant difference between CC and TT genotypes, p = 0.03.

### Logistic regression analysis

Logistic regression analysis was performed to ascertain the effects of selected significant risk factors in the study on likelihood that patients have PCAD.

It showed that age, duration of hypertension and duration of diabetes were not associated with PCAD, *p* = 0.17, 0.6 and 0.1 respectively, while other significant factors remained associated with PCAD (**Table 4**).

**Table 4:** Logistic regression analysis of the significant risk factors for PCAD.

Factor	OR	95% CI	*P* value
LDL-cholesterol	1.7	1.6…2.9	0.018
Triglycerides	0.07	0.01…0.42	0.004
Hcy Level	0.6	0.5…0.8	0.03
T allele	0.19	0.08…0.32	0.02
HDL-cholesterol	1.2	0.96…3.0	0.04
Smoking	0.2	0.1…0.7	0.02
Duration of Hypertension	0.9	0.7…1.1	0.6
Age	0.96	0.95…1.2	0.17
Duration of Diabetes	0.9	0.85…1.0	0.1
MTHFR polymorphism (TT)	2.9	2.3…3.9	0.001

Only risk factors for PCAD which were significantly different between the two groups were included in the analysis. All these factors remained significant for PCAD, except for age, duration of hypertension and duration of diabetes, which have no significant association with PCAD in Logistic regression analysis.

## Discussion

Worldwide, the frequency of MTHFR gene mutations varies among racial and ethnic groups (28, 29). In Africa, MTHFR gene polymorphism is markedly low (below 10%) for 677 T allele (30, 31). In Europeans and North Americans, the frequency ranges from 10-18%, in contrast, the frequency of T allele is markedly high among Chinese populations at around 45% (32). In our study, the mutant T allele frequency was 36% in cases and 8% in controls (**Table 2**). In a study done on Zambian population, the T allele frequency was 1%, and no subject had the TT genotype (31). However, it is known that Sudanese population have a high level of genetic diversity and therefore are unlikely to be representative of African populations south of the Sahara (33, 34).

The frequency of the T allele was significantly different between T2DM patients with and without CAD, *p <*0.001. This finding was consistent with other findings done in diabetic patients from different populations (30, 35).

Elevated homocysteine level (hyperhomocysteinemia) seems to predict cardiovascular events among diabetic patients (6, 36–41). However, many other studies failed to establish the connection and claimed that hyperhomocysteinemia is an innocent bystander among diabetic patients (42, 43). The genetic basis of hyperhomocysteinemia has also been known (44, 45) and MTHFR polymorphism MTHFR 677T allele has been associated with high homocysteine levels (46–48), while others failed to detect such a relationship (49, 50). Ethnicity differences could be a responsible parameter for at least some of these controversial results (28, 29). Hcy levels are also influenced by various environmental factors and, vitamin B_12_ and folate (51, 52). The limitations of this study include the lack of data on drug treatments, not measuring lipoprotein (a) and that folate status and vitamin B_12_ levels were not measured.

Plasma homocysteine levels are influenced by age, diet, genetic background and the use of medications like metformin and fibrates which are used frequently in T2DM patients (53). In the presence of these confounding factors, a single test of plasma homocysteine, in a relatively small number of study subjects, cannot provide enough information for us to establish a causal relationship between homocysteine and PCAD. In addition, the participants in the study came from one city capital Khartoum and might not be representative of the whole country. We therefore cannot recommend universal measurement of homocysteine levels in our T2DM patients especially that homocysteine test is relatively expensive for our patients who are mostly working class. However, health professionals are encouraged to advise patients with T2DM to increase intake of vegetables and fruits, within a balanced diabetic diet. Improvement of lifestyle factors like cessation of smoking, physical exercise and maintaining ideal body weight will decrease the overall risk for cardiovascular disease and may have a positive effect on plasma total homocysteine levels (54).

In conclusion, homocysteine levels are increased in our T2DM with PCAD compared to diabetic patients without CAD. The increase in the homocysteine levels is associated with T allele in MTHFR gene, presence of T allele will increase risk for CAD significantly.

Our study suggested that hyperhomocysteinemia is an important factor for PCAD in our patients with T2DM and more studies are required to confirm these findings and to study the effect of nutritional advice and supplementation with the relevant vitamins on homocysteine levels and prevention of PCAD.

## Data Availability

The raw data supporting the conclusions of this article will be made available by the authors, without undue reservation.
